# The Synthesis and Thermoelectric Properties of the n-Type Solid Solution Bi_2−x_Sb_x_Te_3_ (x < 1)

**DOI:** 10.3390/ma16175941

**Published:** 2023-08-30

**Authors:** Amélie Galodé, Tristan Barbier, Franck Gascoin

**Affiliations:** Laboratoire CRISMAT, ENSICAEN, UNICAEN, CNRS Normandie Université (UMR 6508), 14280 Caen, France; amelie.galode@ensicaen.fr (A.G.); tristan.barbier@ensicaen.fr (T.B.)

**Keywords:** Bi_2_Te_3_, Bi_2−x_Sb_x_Te_3_, n-type, thermoelectric, mechanical alloying, SPS

## Abstract

Commercial Peltier cooling devices and thermoelectric generators mostly use bismuth telluride-based materials, specifically its alloys with Sb_2_Te_3_ for the p-type legs and its alloys with Bi_2_Se_3_ for the n-type legs. If the p-type materials perform with zT well above the unity around room temperature, the n-type counterpart is lacking efficiency in this temperature range, and has the disadvantage of containing selenium. Indeed, despite the fact that selenium is not environmentally benign and that its handling requires precautions, the use of selenium does not facilitate the optimization of thermoelectric performance at or around room temperature, as the presence of selenium results in a larger band gap. In this study, we investigate the feasibility of a selenium-free n-type (Bi, Sb)_2_Te_3_ using a simple two-step process: mechanical alloying synthesis followed by spark plasma sintering. All the members of the solid solution Bi_2−x_Sb_x_Te_3_ with x < 1 are n-type materials, with zTs between 0.35 and 0.6. The zT is maximized at lower temperatures with an increasing Sb content, which is proof that the band gap is reduced accordingly. We also show here that an edge-free sintering process considerably improves thermoelectric performance.

## 1. Introduction

With the highest thermoelectric figure of merit (zT) near ambient temperature, bismuth telluride materials are widely used near room temperature, whether for temperature regulation or waste heat recovery [1]. Adequate or better-performing p-types are solid solutions of (Bi, Sb)_2_Te_3_, while n–types are solid solutions of Bi_2_(Se, Te)_3_. The technology used to make devices with Bi_2_Te_3_-based materials is very advanced and has remained the same for the past 60 years [2,3]. Nevertheless, efforts to develop a better performing Bi_2_Te_3_ material are still ongoing, and have led to numerous reports of electronically and thermally tailored Bi_2_Te_3_ materials with higher figures of merit [4,5,6,7,8].

The electronic properties of Bi_2_Te_3_ are well known to depend on the nature and the concentration of defects that, in turn, depend greatly on the composition (including dopants or not) [9] and on the synthesis and processing conditions. For instance, in the simple binary Bi_2_Te_3_, the ability to control the antisite defect Bi_Te_ is crucial, where Bi replacing a Te on an anion site produces a hole, and Bi^3−^ accepts one more electron than Te^2−^, in agreement with Bi-rich Bi_2_Te_3_ being p-type. On the contrary, if it is made in Te-rich conditions, electron donor antisite Te_Bi_ defects will prevail, thus leading to n-type conduction. In addition to exemplifying the meticulous care and precision that must be employed to produce a compound, property-wise, in a reproducible way, it also emphasizes the trickiness of working with ternary or quaternary derivatives of Bi_2_Te_3_, because competing and/or counteracting effects will very often lead to inhomogeneous materials with drastically different properties.

While for p-type legs, the compositions and properties of (Bi, Sb)_2_Te_3_ are well known and established, it seems that more work has recently been devoted to obtaining a competing n-type counterpart [7,10,11,12]. Indeed, if the state-of-the-art n-type leg appears to have the composition Bi_2_Te_2.7_Se_0.3_, it still has a lower zT than the p-type leg, and because it contains selenium, it demands more precautions to synthesize; moreover, it has mechanical properties that are different from those of the p-leg, and does not behave as well as p-type legs when it comes to soldering or brazing. Thus, for all these reasons, finding n-type Bi_2_Te_3_ that does not contain selenium is clearly appealing.

Reports of n-type are numerous; however, the best-performing compositions always contain a sizable percentage of selenium, and the process associated with the manufacturing of the material is sometimes very complex. This includes off-stoichiometric nominal composition [5,6,7,8,9,10,11,12,13], multiples, hot pressing and/or forging [6,14,15,16] with the addition of inclusions or nano-inclusions [12,17,18], and micro or nanostructure engineering [19,20,21,22], all of these being rather beneficial in the pure performance of the material, but being difficult in the mass production of the material, which requires rather simple and efficient processes. Interestingly, 25 years ago, n-type conduction in the Bi–Sb–Te ternary system for the compositions Bi_1.4_Sb_0.6_Te_3_ and Bi_1.6_Sb_0.4_Te_3_ was reported, whether or not an excess of tellurium was added during the mechanical alloying synthesis steps [23]. This was also confirmed more recently [24] by a study showing that the materials Bi_2-x_Sb_x_Te_3_, produced using a rather lengthy and complex multi-step synthesis that includes melting, grinding, densifying and hot deformation are p-type, for x ≤ 1.3. In this work, we aim to provide a simple way to make n-type (Bi, Sb)_2_Te_3_ using mechanical alloying followed by a short SPS densification, thereby rendering it a suitable way to scale up production. In this work, we will also show that modification of the synthesis and processing parameters can lead to sizable variations in transport properties. This study opens the door to further optimization of selenium-free n-type bismuth tellurides, toward the replacement of the ancient Bi_2_Te_3−x_Se_x_.

## 2. Materials and Methods

### 2.1. Synthesis and Densification 

The title compounds Bi_2−x_Sb_x_Te_3_ (0 ≤ x ≤ 0.9) were prepared by using elemental chunks (all from Alfa Aesar, Haverhill, MA, USA, and used as received) of bismuth (needles, 99.99%), tellurium (shots, 99.99%), and antimony (shots, 99.99%) as precursors. The elements were weighed to obtain the desired stoichiometry. Typically, 12 g of mixture was loaded in a 20 mL tungsten carbide ball mill jar, containing 7 tungsten carbide of 10 mm diameter balls. The mixture was then subjected to mechanical alloying for a short period of 180 min, divided into 30 cycles of 5 min each at 600 rpm in a Fritsch Pulverisette 7 premium line apparatus (Fritsch Idar-Oberstein, Idar, Germany). The obtained powder was compacted using an SPS (spark plasma sintering) FCT HP D 25/1 process in a graphite die (CarbonLorraine, Gennevilliers, France) of 15 mm diameter using a pressure of 28 MPa with a 30 min dwelling at a temperature of 723 K. The resulting cylinders had densities higher than 95% of theoretical ones. For the sake of studying the influence of different synthetic and processing parameters on transport properties, the samples were also produced with a mechanical alloying step performed on 24 g (45 mL/16 balls) of precursors instead of 12 g, and the samples were densified using an SPS dwell time extended to 90 min.

### 2.2. Transport Property Measurements

The resistivity and Seebeck coefficient were measured with an ULVAC ZEM-3 apparatus using the four-probe method and the differential method, respectively. Measurements were made on bars with approximate dimensions of 2 × 2 × 10 mm^3^ between room temperature and 450 K under the partial pressure of 0.1 atm of helium. Thermal diffusivity was determined with a flash laser using an LFA457 device under 20 mL/min nitrogen flow. Samples were 6 × 6 mm^2^ squares with a thickness of about 1 mm. Heat capacity was calculated with Dulong–Petit approximation and used for the determination of thermal conductivity. Evidently, all properties were measured in the same direction, namely the “in-plane” direction, in other words, perpendicularly to the pressing direction.

Hall coefficients were measured at 300 K using a Physical Properties Measurements System (PPMS–Quantum Design) using the same sample used for high-temperature measurements. Electrical contacts were manually made via 0.025 mm diameter indium wires connected to the polycrystalline sample for the four-probe technique. Hall coefficients R_H_ were obtained from a fit of the Hall resistance versus the magnetic field, which was swept from −70 to +70 kOe, and the Hall voltage was determined as VH = 1/2 [V(+H) − V(−H)], where V is the voltage across the Hall terminals. The mobility is evaluated using R_H_ divided by the electrical resistivity.

### 2.3. Characterization

After densification using SPS, small pieces of the dense “pucks” were manually ground before being inspected by X-ray powder diffraction using a Phillips X-Pert Pro PANalytical diffractometer with a K_α1_/K_α2_ radiation (λ = 1.540598 Å and 1.544426 Å, respectively). X-ray diffraction data have been collected from 5 to 100° with a step size of 0.011° and a step time of 2 s. A diffraction pattern of a standard LaB_6_ powder has also been registered under the exact same conditions to obtain the instrumental broadening of the PANalytical diffractometer. The angular dependence of the full width at half maximum (FWHM) of the diffraction peak obtained for this standard was chosen as Caglioti type [FWHM(θ) = (U tan^2^ θ + V tan θ + W)^1/2^, where U, V, and W are refinable parameters [25,26]. The resulting Lorentzian and Gaussian FWHM were added to the instrumental parameters file (Instrumental Resolution Function: *.irf file). The full profile fitting refinements were carried out via the Rietveld method, using the FullProf program [27]. The peak shape was corrected from the instrumental broadening using the aforementioned *.irf file and then modeled with the Thompson–Cox–Hasting (TCH) pseudo-Voigt peak profile function (NPR = 7) [18]. Regarding the structure refinement, systematic error corrections (zero-point shift and asymmetry) were applied, and the background points were manually selected. With respect to the crystallographic structure, the lattice parameters, atomic positions, and isothermal temperature factors (B_iso_) were also refined (the occupancy of all atoms was fixed to 100%, except for elements occupying the same crystallographic site (e.g.,: Bi_2−x_Sb_x_), where the occupancy rates used were those previously calculated theoretically.

## 3. Results

**Figure 1:**
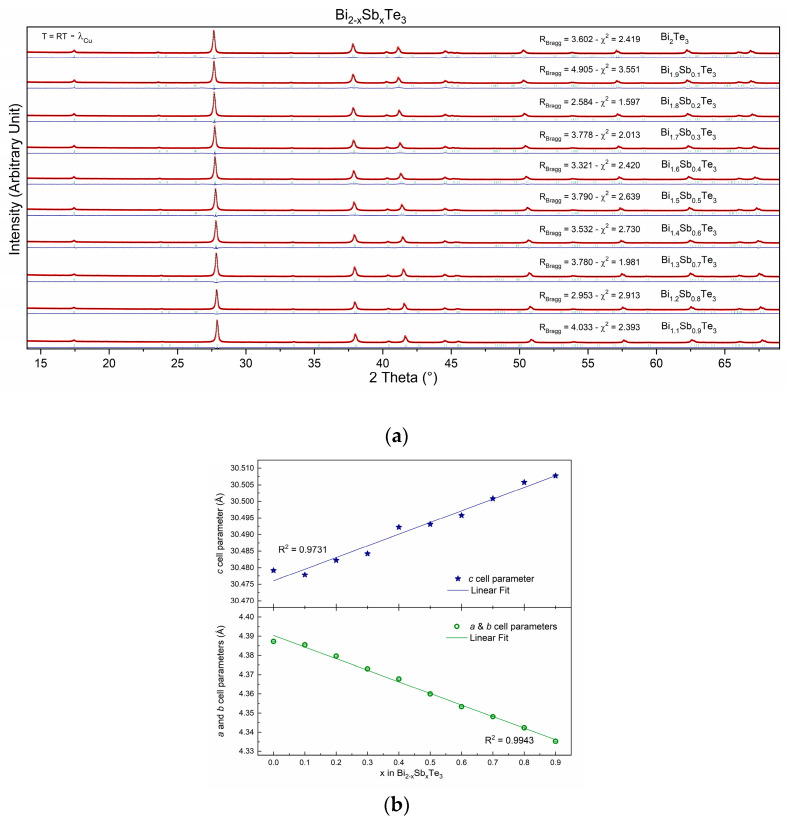
(**a**) Powder X-ray diffraction patterns, recorded at room temperature, of the Bi_2−x_Sb_x_Te_3_ series, where x varies from 0 to 0.9. (**b**) Cell parameters’ evolution with Sb content (x into Bi_2−x_Sb_x_Te_3_).

Some 10 members of the solid solution Bi_2−x_Sb_x_Te_3_ for x varying between 0 and 0.9 were prepared following the exact same recipe (see experimental part). This is indeed mandatory to be able to compare the transport properties of the samples as it is well known that different preparation methods will, most of the time, lead to different properties, as will be exemplified in the second part of this manuscript. This also means that it is rather difficult to compare the results obtained by different research groups, as the techniques or apparatus used are never always the same from one lab to another, even without acknowledging the “human factor”. Powder X-ray diffraction studies have been performed after the final sintering step, i.e., just before the physical property measurements. The X-ray diffraction patterns, recorded at room temperature, of all samples synthesized in the series Bi_2−x_Sb_x_Te_3_ with x varying from 0 to 0.9 are gathered in Figure 1a. All patterns have been refined using the R$\bar{3}$m (n°166) space group. It should be mentioned that all peaks (from all patterns) have been correctly modeled by the space group, and no additional peaks are observed on the diagrams, thus confirming the high purity of the samples (also confirmed by the reliability factors displayed in Figure 1a).

Rietveld refinements were used to calculate the cell parameters of the various compounds. The results, in graphical form (Figure 1b), show two behaviors, each following Vegard’s law, thus confirming the successful substitution of Sb at the Bi crystallographic site. The first is for parameters a and b (a = b = 4.3 Å), and the second is for the c cell parameter (c = 30.4 Å). Indeed, as can be observed through Figure 1b, the a and b cell parameters decrease while the c cell parameter increases as the Sb content (x) increases (into Bi_2−x_Sb_x_Te_3_). Such phenomena may be attributed to the different atomic sizes and electronegativities of Bi and Sb. Indeed, Bi is a larger atom than Sb (r(Bi^3+^ − VI) = 1.03 Å and r(Sb^3+^ − VI) = 0.76 Å), so it takes up more space in the unit cell [28,29]. This is why the a and b cell parameters decrease as the Sb content increases [28]. Sb (χ = 2.05), on the other hand, has a higher electronegativity than Bi (χ = 2.02), so it attracts the electrons in the Te-Te bonds more strongly. This causes the Te atoms to be pulled further apart, which is why the c-cell parameter increases [30,31].

**Figure 2:**
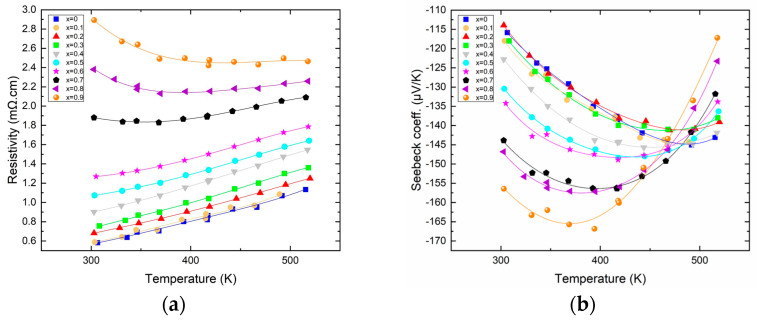
Resistivity ((**a**)—**left**) and Seebeck ((**b**)—**right**) of Bi_2−x_Sb_x_Te_3_ as a function of temperature.

Figure 2a shows the variation in electrical resistivity as a function of temperature for all the members of the solid solution. The electrical resistivity increases nicely with temperature, and the first members (for x ≤ 0.6) exhibit resistivities reminiscent of those of metals with a linear increase with increasing temperature (0.24 mΩ·cm per 100 K); meanwhile, for higher antimony content, an onset of semi-conducting behavior is visible, which is the first sign of the transition from n to p-type that will occur if the Sb content is increased further. The resistivity of the samples also increases with antimony content, with a factor of 5 between the most resistive (x = 0.9) and the least resistive (x = 0) at ambient temperature. Figure 2b shows the variation in the Seebeck coefficient as a function of temperature for all the members of the solid solution. The general trend is that the Seebeck increases in absolute value with increasing antimony content along the solid solution. In more detail, it is remarkable that for x between 0 and 0.3, the Seebeck remains virtually unchanged but starts increasing substantially for a higher content of antimony, with the highest Seebeck coefficient being about −170 µV/K for x = 0.9. Furthermore, the temperature at which the thermopower passes through a maximum decreases with increasing antimony content. Indeed, for low Sb content, the Seebeck is maximized at around 460 K, while for the highest Sb content, the maximum Seebeck is obtained at about 350 K, in good agreement with the fact that with increasing Sb content, the band gap is reduced, hence the early (temperature-wise) apparition of a bipolar effect. 

The variations in both the resistivity and the Seebeck are reminiscent of what is expected when the carrier concentration varies along the solid solution. However, if the carrier concentration measured around 300 K indeed shows a decrease with increasing Sb content, this decrease is only very small, roughly about 2.0 × 10^19^ cm^3^ from Bi_2_Te_3_ (x = 0) to Bi_1.1_Sb_0.9_Te_3_. Evidently, such a small variation cannot explain the difference in electronic transport properties. The origin of the increase in resistivity is rather found in the large decrease in mobility. Indeed, the mobility measured at 300 K varies monotonically from 160 cm^2^/Vs for x = 0 to 40 cm^2^/Vs for x = 0.9, this being associated with the increased alloy scattering. This factor 4 in the decrease in mobility added to the slight decrease in carrier concentration account well for the factor of about 5 found in between the resistivity of the two extremities of our solid solution. As for the variation in the Seebeck coefficient, it is mostly attributed, as demonstrated elsewhere, to the increased valley degeneracy, together with a slight contribution of the diminishing charge carrier concentration [15].

**Figure 3:**
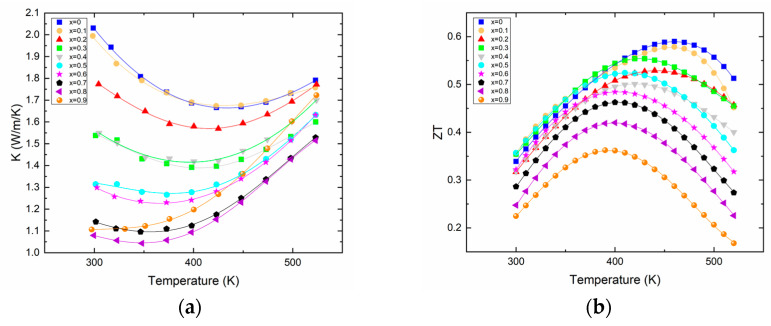
Thermal conductivity (**a**) and thermoelectric figure of merit ZT (**b**) of Bi_2−x_Sb_x_Te_3_ as a function of temperature.

The thermal conductivity (Figure 3a) evidently benefits from the Bi/Sb substitution as shown by its constant decrease with increased Sb content. It is noteworthy that the variation of the lattice component of the thermal conductivity κ_lat_ (see Supplementary Information Figure S1) does not follow any particular trend, as the “beneficial” alloy scattering (due to the substitution of Bi by Sb) that should lower the κ_lat_ is competing with the substitution of a heavy bismuth by a lighter antimony, this being detrimental to the κ_lat_. The actual value of the total thermal conductivity decreases by about a factor of 2 at 300 K. As expected from the electrical transport properties, the bipolar effect is s easily visible for all the different compositions. For each sample, the minimum thermal conductivity is basically reached at a temperature where the Seebeck coefficient maximizes. Overall, the minimum thermal conductivity reaches a value around 1 W/(m·K) at room temperature, a typical value for this family of compounds whose thermal conductivity rarely falls down to lower values, even for materials with a record high thermoelectric figure of merit, or materials prepared by more complex processes [13,15,24].

The substitution of bismuth for antimony has clear effects on electronic and thermal transport, as explained earlier; however, it does not improve the absolute value of the thermoelectric figure of merit. Actually, with the synthesis and process we used, the highest figure of merit is obtained for the material that does not contain any antimony, which is about 0.6 at 460 K for Bi_2_Te_3_, as shown in Figure 3b. Rather, the alloying with Sb leads to lower ZT_max_ that is reached at lower temperatures. This is due to the onset of the bipolar effect that occurs at temperatures decreasing with increasing antimony content. This result is quite in opposition with the results reported recently on such solid solutions; however, as already said, the processes used in both studies are different, and therefore can lead to differences in transport properties that are amplified in the calculation of the ZT [24]. This is even less surprising when considering that in the ternary system Bi–Sb–Te, tellurium vacancies will coexist with antisite defects Bi_Te_ or Sb_Te_; these two types of defects having counteracting effects, inhomogeneous materials can easily be formed when the process does not allow the system to reach an equilibrium state.

To exemplify the influence of the process on the transport properties, three samples (named S1, S2, S3) of the same composition, Bi_1.5_Sb_0.5_Te_3_, were made with different synthesis conditions and/or different sintered methods. Table 1 details the different processes.

The sample S1 is made using the same process used for all the samples discussed in the first part of the manuscript; this is basically how Bi_2_Te_3_-based samples are typically prepared in all the studies reported by our research group, so that comparisons are coherent. S2 is prepared using the same mechanical alloying parameters, but is sintered using a 450 °C plateau three times longer than S1. S3 is a 24 g sample compared to S1 and S2 that are 12 g batches. The main difference in S3 is that fact that it undergoes a first sintering, the same way that S2 does, followed by a second hot pressing, this time using a larger pressing die; thus, this double-pressing can be assimilated into hot forging (more details can be found elsewhere) [15].

Comparing the transport properties of S1 and S2 leads to the quick conclusion that the duration of the plateau at which the samples are sintered does not modify anything; indeed within the measurement incertitude, the Seebeck coefficient, electrical resistivity, and thermal resistivity of both samples are basically the same. Evidently, if the electrical resistivity and the total thermal conductivity are the same, the electronic and the lattice components of the thermal conductivity are the same. Room temperature Hall effect measurements reveal a small decrease in the carrier concentration from S1 (6.3 × 10^19^ cm^−3^) to S2 (5.4 × 10^19^ cm^−3^) and a slight increase in the carrier mobility from S1 (70 cm^2^/(Vs)) to S2 (90 cm^2^/(Vs)). These variations may be associated with the longer time spent at 450 °C, this annealing being in turn responsible for a probable slight grain growth, even if the difference in grain size between the two samples cannot be clearly seen via electron microscopy (see SEM pictures in the supplementary information). However, these two rather minor modifications do not lead to any sizable variation in properties.

The sample S3 was made in order to assess the effect of hot forging on the properties. Thus, 24 g of powder had to be prepared so that the final sample was large enough to be measured in the correct direction. Accordingly, there is a ratio of 2.25 between S1 and S3 in terms of the volume of the milling jar and in terms of the number of grinding balls, but only a ratio of 2 between the mass of precursors, 12 g for S1 and 24 g for S3. S3 is first densified the same way S1 is densified, the resulting 15 mm diameter dense cylinders obviously being twice longer from S1 to S3, as twice the amount of powder is processed. Remarkably, the density of the pucks is basically always the same after this sintering step, at 7.3 g/cm^3^, about 98% of the theoretical density. Following this first densification, the S3 15 mm diameter puck is inserted in a 20 mm diameter graphite pressing die and subjected to a second densification. This process is utilized in order to try to texture the sample, as described elsewhere [15]. Evidently, as the sample is heated a second time, a grain growth is also expected, especially since the size difference between the puck and the die allows a certain liberty of movement of the grain that will be deformed, aligned, and could grow more easily than if they were constrained. The influence of this double-pressing is clearly visible on the transport properties, as the resistivity of the S3 sample drops by about 25% compared to the samples pressed only once; as a consequence, the electronic component of the thermal conductivity is increased, whereas its lattice component is not changed, thus resulting in a net slight increase in the total thermal conductivity of about 0.1 W/(m·K), which is less than 10%, compared to S1. In parallel, since the Seebeck coefficient is not modified, the gain in the ZT is thus sizable, as shown in Figure S3 and corroborating what we have previously reported on n-type Bi_2_(Te, Se)_3_. The powder X-ray diffraction pattern of the S3 sample is shown in Figure S3, and displays a noticeable preferred orientation in the perpendicular direction compared to the parallel direction of the pellet. The elongated grains tend to align with their planes perpendicular to the pressing direction; therefore, noteworthy enhancement of the (00l) peak intensities is easily observed due to the two-dimensional morphology of the grains in the puck.

## 4. Conclusions

In this contribution, we have demonstrated the feasibility of making selenium-free n-type Bi_2_Te_3_ materials by substituting a small amount of bismuth with antimony. The compounds are simply made by combining mechanical alloying and spark plasma sintering. The transport properties along the solid solution vary smoothly with increasing antimony content in accordance with a diminishing band gap. Along the solid solution, the greater the antimony content, the smaller the temperature at which the zT is maximum. In a second step towards improvement, we also show that a simple second hot pressing, as expected, results in a clear preferred orientation of the grains, leading to an improved thermoelectric figure of merit. This work opens the route to further development of Se-free n-type bismuth telluride-based materials.

## Figures and Tables

**Table 1:** Details on the synthesis and sintering parameters for samples of the same composition: Bi_1.5_Sb_0.5_Te_3_. Sample S1 is made using the recipe used for all the samples of the solid solution described in the first part of the manuscript. All the samples are sintered via SPS into 15 mm diameter pucks. Sample S3 is the only one that was hot-pressed a second time, the second hot-pressing being done into a 20 mm inner diameter die. The density of all samples is virtually the same and higher than 95% of the theoretical density. All the syntheses are performed with tungsten carbide balls and jars. For every SPS cycle, the temperature was raised to 450 °C in 18 min, and cooling was performed in 18 min.

Sample	Mass Synthesized	Ball Milling Parameters	SPS Parameters	Second SPS Parameters
S1	12 g	Jar volume: 20 mL	Die size: 15 mm OD Dwell T°: 450 °C Dwell time: 30 min	n/a
		7 balls 10 mm diameter		
		Speed: 600 rpm 30 cycles of 5 min		
S2	12 g	Jar volume: 20 mL	Die size: 15 mm OD Dwell T°: 450 °C Dwell time: 90 min	n/a
		7 balls 10 mm diameter Speed: 600 rpm 30 cycles of 5 min		
S3	24 g	Jar volume: 20 mL	Die size: 15 mm OD Dwell T°: 450 °C Dwell time: 30 min	Die size: 20 mm OD Dwell T°: 450 °C Dwell time: 3 min
		16 balls 10 mm diameter		
		Speed: 600 rpm		
		30 cycles of 5 min		

## Data Availability

Data are contained within the article or as Supplementary Materials.

## Supplementary Materials

The following supporting information can be downloaded at: https://www.mdpi.com/article/10.3390/ma16175941/s1, Figure S1: Lattice component of the total thermal conductivity of _Bi2-x_Sb_x_Te_3_ as a function of temperature. Figure S2: SEM picture of Bi_1.5_Sb_0.5_Te_3_ prepared via three different methods. Figure S3: powder diffraction patterns of Bi_1.5_Sb_0.5_Te_3_ showing a preferred orientation after a hot forging treatment. Figure S4: transport properties and thermoelectric figure of merit of Bi_1.5_Sb_0.5_Te_3_ prepared via different processes. Table S1: Other crystallographic parameters deduced by Rietveld modeling. Atomic coordinates Te1 (0, 0, 0)–Te2 (0, 0, z)–Bi1 (0, 0, z’).
