# Supplementary material for: The Synthesis and Thermoelectric Properties of the n-Type Solid Solution Bi2−xSbxTe3 (x < 1)
Source: Materials (Basel). 2023 Aug 30;16(17):5941. doi: 10.3390/ma16175941 (PMC10488999; doi:10.3390/ma16175941)
Supplement: Supplementary file 1 [file materials-16-05941-s001.zip › materials-2542078-supplementary.pdf]

Supplementary materials

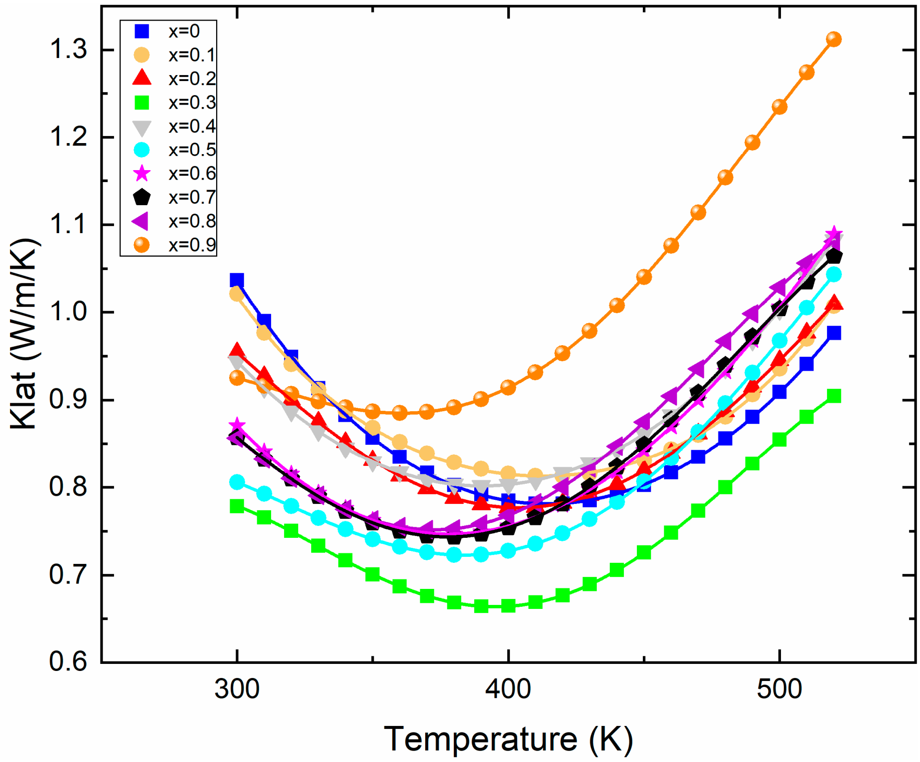

Figure S1 : Lattice component of the total thermal conductivity of $Bi_{2-x}Sb_xTe_3$ as a function of temperature

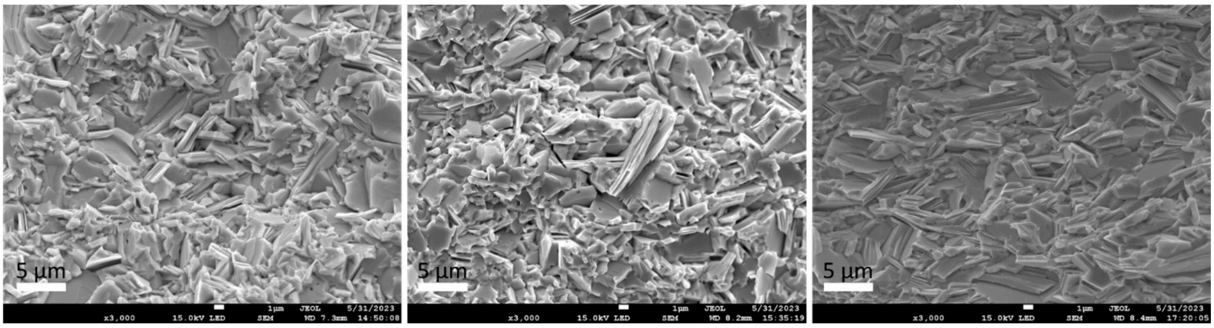

Figure S2 : SEM Picture of broken ingots of S1 (left), S2 (center) and S3 (right), using the same magnification, looking perpendicularly to the pressing direction. No clear difference can be seen between S1 and S2. The S3 sample shows the onset of slight alignment of the grains.

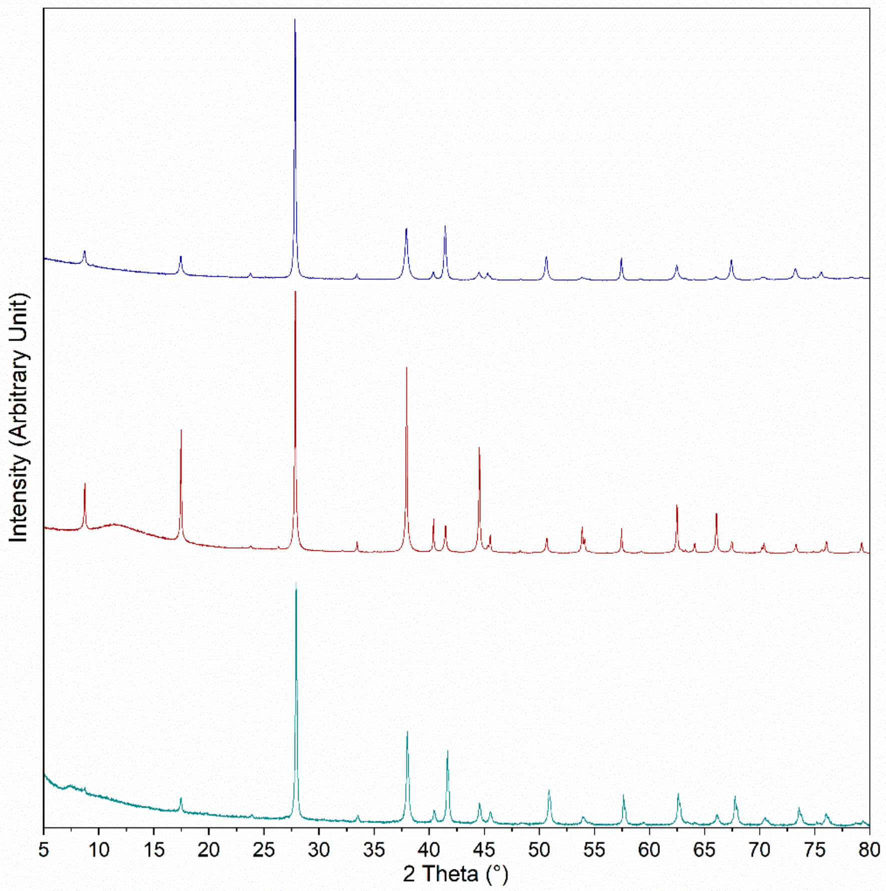

Figure S3 : powder diffraction patterns of $Bi_{1.5}Sb_{0.5}Te_3$ (R-3m pace group, Te1 (0, 0, 0) – Te2 (0, 0, z) – Bi/Sb (0, 0, z')) acquired in three different ways, top: height of the cylindrical puck (parallel to the pressing direction) middle: surface of the cylindrical puck (perpendicular to the pressing direction); bottom: crushed powder after SPS

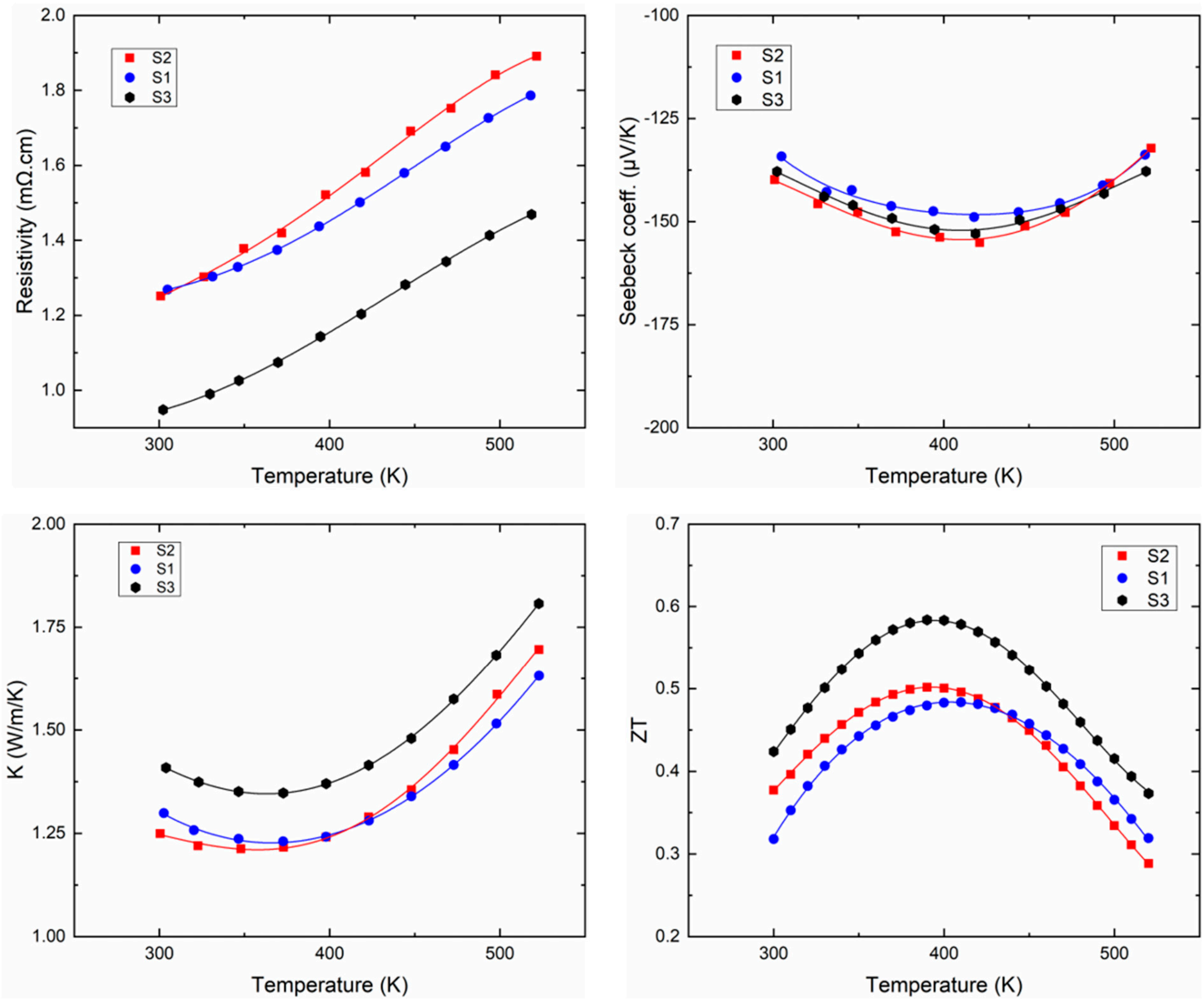

Figure S4: Transport properties and thermoelectric figure of merit of the samples S1, S2 and S3.

Table S1: Other crystallographic parameters deduced by the Rietveld modeling. Atomic coordinates Te1 (0, 0, 0) – Te2 (0, 0, z) – Bi1 (0, 0, z')

	Bi_2Te_3	$Bi_{1.9}Sb_{0.1}Te_3$	$Bi_{1.8}Sb_{0.2}Te_3$	$Bi_{1.7}Sb_{0.3}Te_3$	$Bi_{1.6}Sb_{0.4}Te_3$	$Bi_{1.5}Sb_{0.5}Te3$	$Bi_{1.4}Sb_{0.6}Te_3$	$Bi_{1.3}Sb_{0.7}Te_3$	$Bi_{1.2}Sb_{0.8}Te_3$	$Bi_{1.1}Sb_{0.9}Te_3$
	Space group: R-3m (n°166)									
a	4.38726(4)	4.38550(5)	4.37964(5)	4.37301	4.36780(6)	4.35995(5)	4.35335(5)	4.34810(5)	4.34228(4)	4.33529(5)
c	30.4802(4)	30.4738(4)	30.4822(4)	30.4842(4)	30.4922(4)	30.4931(4)	30.4948(4)	30.5009(4)	30.5058(4)	30.5004(4)
Te1 - B_{iso}	0.90(9)	1.32(10)	1.35(9)	1.16(9)	1.29(10)	0.94(9)	1.34(9)	0.49(9)	0.53(8)	1.5(1)
Te2 - z	0.20911	0.2093	0.20954	0.21024	0.2094	0.21021	0.21053	0.21022	0.21037	0.20959
Te2- B_{iso}	1.1(1)	1.5(1)	1.7(1)	1.9(1)	1.5(1)	1.0(1)	1.6(1)	0.41(10)	0.86(9)	1.2(1)
Bi1/Sb1 – z'	0.40056	0.40074	0.40057	0.40035	0.40042	0.39988	0.39987	0.39972	0.39986	0.3998
Bi1/Sb1 - Biso	1.29(6)	1.52(6)	2.10(7)	2.02(7)	2.00(8)	1.31(7)	1.97(7)	0.64(6)	0.67(5)	1.19(6)
Average apparent size (anisotropy):	540.2300 (46.9600)	526.7800 (51.9100)	514.4400 (60.1600)	507.9500 (49.7200)	504.1300 (43.3200)	528.1800 (51.2600)	483.7500 (51.4900)	527.4200 (30.0400)	583.2400 (25.5600)	514.6200 (42.4900)
